# Family Relationships Under Work From Home: Exploring the Role of Adaptive Processes

**DOI:** 10.3389/fpubh.2022.782217

**Published:** 2022-03-09

**Authors:** Hongyue Wu, Q. Chelsea Song, Robert W. Proctor, Yunfeng Chen

**Affiliations:** ^1^Construction Automation, Robotics, and Ergonomics (CARE) Lab, School of Construction Management Technology (SCMT), Purdue University, West Lafayette, IN, United States; ^2^Department of Psychological Sciences, Purdue University, West Lafayette, IN, United States

**Keywords:** work from home (WFH), family relationships, adaptive processes, work-life balance, Vulnerability-Stress-Adaptation (VSA) model

## Abstract

Work-from-home (WFH) influences both work and life, and further impacts family relationships. The current study explored the impacts of WFH on family relationships during the COVID-19 pandemic and identified effective adaptive processes for maintaining family relationships under WFH. Using the Vulnerability-Stress-Adaptation (VSA) model, the study examined the roles of adaptive processes (spending time with family members and balancing work and life) and demographic differences (gender, age, marital status, and education level) in the relation between WFH and family relationships. Path analysis results based on an online survey (*N* = 150) suggested that, overall, WFH improved family relationships through proper adaptive processes. WFH had a positive relation to time spent with family members, and this relation was especially salient for workers with lower education levels. While there was no statistically significant overall relation between WFH and work-life balance, older workers tended to engage in increased work-life balance during WFH. Both adaptive processes were positively related to family relationship quality. The findings advance the understanding of family relationships and WFH and provide practical recommendations to enhance family relationships under WFH.

## Introduction

Many workplaces are adopting work-from-home (WFH) arrangements, especially since the COVID-19 pandemic. In the United States (U.S.), stay-at-home orders were widely implemented (1) during the pandemic to control the spread of the virus (2), leading many workplaces to rapidly shift to WFH arrangements (3, 4). Over 34% of workers in the U.S. shifted to WFH since March 2020 (5, 6), and over 148,383 individuals continued to work from home in January 2021 (7). Some workplaces are considering implementing the WFH arrangements permanently, even after the pandemic (8).

WFH causes changes to both work and life environments, impacting family relationships (9, 10). Family relationships refer to relationships with spouses, children, parents, and siblings (11), and are among the most critical personal relationships (12). Family relationships have critical impacts on wellbeing and mental health (13–15). Strong family relationships provide crucial social support (11) that could mitigate psychological problems including anxiety and loneliness (16). As a rapidly growing future work arrangement, WFH may have a widespread impact on family relationships and affect the wellbeing and mental health of many individuals (17). However, previous studies have suggested many variations in the relation between WFH and family relationships. Under WFH, some individuals experienced more family support (18), and domestic violence crimes fell by 8.7% (19). On the contrary, other individuals experienced difficulty maintaining personal relationships (including family relationships) during WFH, especially under the COVID-19 pandemic (9, 10). Thus, the current study investigates how WFH influences family relationships and related factors. Specifically, the study aims to provide insight into how individuals adapt to WFH and how such adaptive processes impact their family relationships using the Vulnerability-Stress-Adaptation (VSA) model. By doing so, the study has implications for relationship theories and provides suggestions for facilitating the wellbeing and mental health of workers and their families (17). The findings could help workers with diverse backgrounds (gender, age, marital status, and education level) adapt to WFH and achieve satisfying family relationships.

## Theory and Hypotheses

### Vulnerability-Stress-Adaptation Model

The Vulnerability-Stress-Adaptation (VSA) model illustrates how individuals with different vulnerabilities adapt to stressful events and how that adaptive process influences relationship quality (20). Vulnerabilities (a.k.a., enduring vulnerabilities) refer to “stable demographic, historical, personality, and experiential factors” that individuals bring to the relationships (20) (p. 22). Stressful events describe the circumstances that individuals encounter (e.g., transitions, difficulties, incidents, and any chronic or acute changes in the external environment) (20), which relate to disruptions in cognitive and behavioral control (21). Adaptive processes are the actions that individuals take to contend with stressful events (20), which include problem-solving, cognitive attribution, and dyadic coping (22).

According to the VSA model, under stressful events, individuals with different vulnerabilities embrace different processes to adapt to the stress, which subsequently impacts the quality of their relationships with others (20). Specifically, the VSA model illustrates the following relations. First, external stressful events (e.g., WFH) will trigger individuals to engage in adaptive processes (e.g., spending time with family members and balancing work and life). Second, individuals' vulnerabilities (e.g., demographic differences) influence their abilities to adapt to stressful events and challenges; that is, the effect of the stressful event on adaptive processes could be different for those with different vulnerabilities. Third, the adaptive process further influences individuals' quality of relationships. Finally, the relationship quality will in turn impact individuals' abilities to adapt to stressful events.

The VSA model has been supported in studies of close relationships (e.g., marital relationships, family relationships) under various stressful events. For example, a study concluded that a higher level of depressive symptoms (vulnerability) was associated with increased perceived life stress, less adaptive interaction with their partners (adaptive process; e.g., joint decision-making), and higher marital relationship risk (23). A recent review suggested that individuals with different vulnerabilities (e.g., gender, negative affectivity, neuroticism, depression) reacted to everyday hassles (stressful event) by engaging in different amounts of social withdrawal (adaptive processes), which was associated with differing family relationship qualities (24). In addition, findings based on self-reported data suggested that the vulnerabilities (e.g., internalized homophobia and outness) moderated the effect of perceived daily stress on daily relationship quality (25). The VSA model provides a systematic and dynamic illustration of the factors (both external environment and internal characteristics) and processes that influence family relationships (26) during stressful events.

### Conceptual Model and Hypotheses

**Figure 1:**
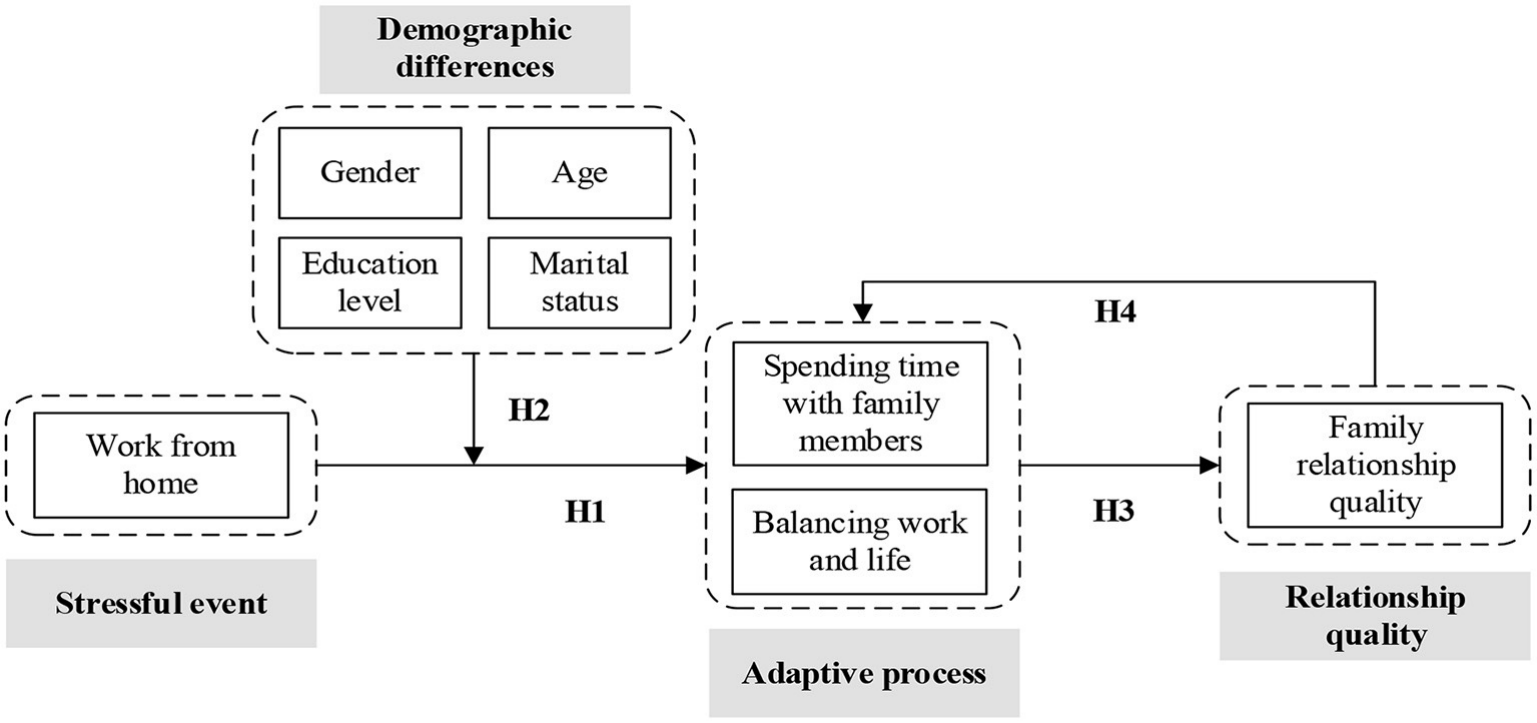
Proposed conceptual model.

The current study uses the VSA model to examine what adaptive processes (i.e., spending time with family members and balancing work and life) will individuals with different demographic backgrounds (gender, age, marital status, and education level) adapt under WFH and how that is related to family relationship quality. Figure 1 shows the proposed conceptual model.

The WFH arrangement is the stressful event examined in the current study. The WFH arrangement is the stressful event examined in the current study. In particular, the study focused on the individuals' WFH experiences during the early stage of the pandemic, when most individuals were new to WFH and needed to adapt to this significantly different work arrangement in a very short period of time (3). This rapid shift to WFH is an unprecedented circumstance and a stressful event that led to acute changes in both work and life environments. During this change, individuals engaged in adaptive processes to maintain (or improve) family relationship quality. In this study, two generally used adaptive processes were considered: (a) spending time with family members and (b) balancing work and life. Family relationship quality largely relies on the communication among family members (12, 27). WFH allows workers to stay at home and spend time with family members, providing an opportunity to enhance communication and further improve family relationships (19, 28, 29). Family relationship quality is also influenced by the balance between work roles and family/life roles (12, 30). Good work-life balance can reduce work-family conflict and contribute to better family relationships (31). Under WFH arrangements, work and life aspects become more inseparable (32, 33). Previous studies showed that, compared to in-person work arrangements, WFH allows workers to have flexible work schedules, less traveling time, and more support from family members, and thus fosters better balance between work and life (18, 33–36). Recent studies of the COVID-19 pandemic suggested that some workers experienced better work-life integration and balance when working remotely (37, 38). On the contrary, other workers faced increased work-life conflict due to the difficulty in distinguishing between work and life during WFH (39, 40), including during the COVID-19 pandemic (41, 42). Overall, more studies supported the positive impact of WFH on work-life balance (33). Therefore, our first Hypothesis suggests that WFH has significant positive associations with two adaptive processes: (a) Workers spend more time with family members during WFH; (b) Workers can balance work and life better during WFH. While spending time with family members emphasizes absolute time with family, balancing work and life focuses on the relative time and attention devoted to life and work.

***Hypothesis 1:*** WFH positively relates to the adaptive processes of (a) spending more time with family members and (b) balancing between work and life.

Demographic differences (or vulnerabilities) such as gender (43–45), marital status (24, 28), age (46, 47), and education level (48, 49) may influence the adaptive processes in which workers engage during WFH. Gender and marital status influence individuals' family engagements. Women and married couples typically have more housework and childcare responsibilities (28, 45, 50), which increases their time with family members, but causes difficulties in balancing work and life during WFH. Age may influence the adaptive process that workers engage in as individuals in different age groups have different experiences and responsibilities in terms of both work and life (46, 47). For example, older workers can spend more time with family members and maintain better work-life balance due to a high level of autonomy (51), and better family embeddedness (52). Educational attainment also influences adaptive processes: highly educated individuals may have more difficulties in maintaining the balance between work and life during WFH (49). Thus, our second Hypothesis suggests that the relations between WFH and adaptive processes will be different among workers with different genders, marital statuses, ages, and education levels, highlighting the moderating effects of the demographic differences.

***Hypothesis 2:*** Demographic differences (gender, marital status, age, and educational level) moderate the association between WFH and adaptive processes.

Adaptive processes further relate to family relationships. Spending time with family members increases the opportunity for communication and interaction, and mitigates the adverse effects of stressful events (46), thus enhancing family relationships (27, 44). In addition, work-life balance can reduce work-family conflict and contribute to better family relationships (30, 31). Thus, our third Hypothesis suggests that the two adaptive processes positively relate to family relationships.

***Hypothesis 3:*** Adaptive processes (spending time with family and balancing work and life) are positively related to family relationship quality.

In turn, the quality of family relationships informs the adaptive processes, creating a feedback loop, and thus a reciprocal relation between adaptive processes and family relationship quality. During WFH, strong family relationships encourage individuals to spend more time with family members to maintain closeness and receive social support (53). Family relationships also have a significant impact on work and life satisfaction (54), which associates with the balance between work and life. Our fourth Hypothesis assumes that family relationship quality further influences the two adaptive processes.

***Hypothesis 4:*** Family relationship quality further influences individuals' adaptive processes (spending time with family and balancing work and life). There is a reciprocal relation between family relationship quality and adaptive processes.

## Methodology

### Participants and Sample

Participants were workers in the U.S., who were experiencing WFH during the COVID-19 pandemic. Because more than 34% of the U.S. workers transferred to WFH beginning in March 2020 (5), an online survey was distributed between May 7th to May 28th, 2020 to capture the participants' experiences after working from home for at least one or 2 months. In the survey, the participants were first asked to provide demographic information. They were then asked to report their WFH experiences by specifying how many days per week they worked from home, and they responded to key study variables (as discussed below) before and during WFH. Participants were invited through multiple channels. First, recruitment messages were posted on social media such as LinkedIn and Facebook. Second, to engage more participants, individual and customized emails were sent out to contacts identified through social media, university directories, and professional organizations. Specifically, the online survey was distributed to some staff and faculties from 25 universities and contact persons of 12 nationwide professional associations.

The data collection yielded 191 participants. Among them, 10 participants outside the U.S., 26 students, and 5 participants who were laid off or furloughed were removed. The final sample size was 150. Data collection took place in one setting, and all participants were asked to provide two responses for the same set of items (that capture key study variables) for both (a) before WFH and (b) during WFH. 27 responses from participants who did not experience a transition to WFH (i.e., who already worked from home before the pandemic) and 8 responses from participants who never worked from home were removed. In addition, 2 responses with missingness for all variables except for demographic variables were removed. Finally, 263 responses^1^ from 150 participants were used for further analysis.

The 150 participants covered diverse industries, including education (67.33%; e.g., professor, researcher, and teacher/instructor), Architecture, Engineering, Construction, and Operation (AECO; 21.33%), manufacturing (4.00%), professional, scientific and technical services (3.33%), information (1.33%), and others (2.67%). Among the participants, 111 (74.00%) were men and 37 (24.67%) were women. Two participants preferred not to disclose the information. 34 (22.67%) participants were between the ages of 20–30; 46 (30.67%) participants were between the ages of 30–40; 26 (17.33%) participants were between the ages of 40–50; 27 (18.00%) participants were between ages of 50–60, and 17 (11.33%) participants were older than 60 years. Considering marital status, 44 (29.33%) participants were single, while 100 (66.67%) participants were married or lived with an intimate partner. Six participants chose to not provide the information. For education level, 60 (40.00%) participants had doctorate degrees; 58 (38.67%) participants had master's degrees; 27 (18.00%) participants had bachelor's degrees; 5 (3.33%) participants had associate degrees or below.

### Measures

The study examined four major variables: stressful event, demographic differences (vulnerabilities), adaptive process, and family relationship quality. Table 1 summarizes all the items used to measure the variables. The participants also provided qualitative comments relating to these variables.

**Table 1:** Variables examined in the study and items used to measure them.

**Variables**	**Indicators**	**Items**	**References**
Demographic differences	Gender	What is your gender?	(12, 55)
	Age	What is your age?	(12, 46)
	Marital status	What is your marital status?	(12, 46)
	Education level	What is your highest level of education?	(48)
Stressful event	Work from home	How often (days/week) do you work from home before stay-at-home orders, during stay-at-home orders, and after stay-at-home orders?	(2, 16)
Adaptive process	Spending time with family members	Please rate the level to which you agree with the following item before WFH and during WFH along a five-point Likert scale: spending more time with family members	(12, 48, 56, 57)
	Balancing work and life	Please rate the level to which you agree with the following item before WFH and during WFH along a five-point Likert scale: balancing work and life better	(31, 36, 41, 58, 59)
Family relationship quality	Satisfaction-level of family relationships	Please rate your satisfaction level of relationships with family members before WFH and during WFH along a five-point Likert scale.	(22, 55)

#### Stressful Event

The stressful event examined in the current study is WFH. The data were collected in May 2020 and describes the participants' WFH experiences during the early stage of the pandemic, when a majority of the participants were new to WFH and had a very short amount of time to adapt to it. To evaluate the WFH experiences, participants were asked to respond to: “How often (days/week) do you work from home before stay-at-home orders and during stay-at-home orders?” The response to this question indicates the extent to which the participants experienced WFH. In the data analysis, WFH was a dichotomous variable, where 0 indicated regular work (i.e., mainly work in the office or on-site), while 1 indicated WFH.

#### Demographic Differences

The current study examined four demographic differences: gender, age, marital status, and education level. Gender was a dichotomous variable, where 0 indicated women and 1 indicated men. Marital status was also dichotomous, where 0 indicated single and 1 indicated married or lived together. Education level was a categorical variable, where 1 indicated high school or equivalent; 2 indicated some college; 3 indicated associate degree; 4 indicated bachelor's degree; 5 indicated master's degree; and 6 indicated doctoral degree.

#### Adaptive Process

Adaptive process is the action that individuals take to adapt to stressful events (48). The current study focused on the adaptive processes of (a) spending time with family members and (b) balancing work and life. For spending time with family members, participants were asked to respond to the item, “spending more time with family members,” using a 5-point Likert scale, where “1” indicated “strongly disagree” (meaning that the participant did not spend much time with family members), and “5” indicated “strongly agree” (meaning that that the participant spent much time with family members). The wording of this item aligns with previous studies on adaptive processes [e.g., (56)], which measured the process using items including “more family time” and “greater time with families.” For balancing between work and life, the participants were asked to respond to a single item, “balancing work and life better,” using a 5-point Likert scale, where “1” indicated “strongly disagree” (meaning that the participants could not balance work and life), and “5” indicated “strongly agree” (meaning that the participant could reach a balance between work and life). Previous studies also used single items to measure the balance between work and life. Zheng et al. (31) asked participants to rate work-life balance with a 5-point Likert scale, where 1 was “not at all balanced” and 5 was “very balanced.” Palumbo (42) measured work-life conflict using one-item, where 1 indicated “no experiences of work-life conflict” and 5 indicated “frequent experience of work-life conflict.”

#### Family Relationship Quality

Relationship satisfaction is a commonly used subjective measure of relationship quality, which is often measured using single items (55, 60). In this study, a single item with a 5-point Likert-like scale was used to measure the satisfaction level of family relationships, where 1 indicated “very dissatisfied” and 5 indicated “very satisfied.”

### Data Analysis

All the item- and construct-level missing data were imputed using multiple imputation MI; (61). MI was carried out using PROC MIANALYZE in SAS. The procedure imputes the missing data multiple times using regression and randomization, and estimates the pooled results (parameter estimates) across the multiple imputed samples. Descriptive analysis was performed to summarize the demographic information and calculate the mean, and standard deviations. Pearson correlations were estimated using PROC CORR in SAS to examine the relations among variables. Structural equation modeling (SEM), specifically path analysis, was used to test the Hypotheses on the relations among vulnerability (demographic differences), stressful event, adaptive processes, and family relationship quality based on the self-reported data. Path analysis can examine situations that several variables act as chains of influence (62), making it suitable for evaluating the Hypotheses of the current study. The current study aims to examine the effects of each adaptive process rather than their comparison, and thus two separate path models were used to study the two adaptive processes. This practice is consistent with previous studies on the VSA (63). PROC CALIS in the SAS software was used to conduct path analysis (64, 65). Chi-square, degree of freedom (*df*), χ^2^/*df*, standardized root mean square residual (SRMR), goodness of fit index (GFI), root mean squared error of approximation (RMSEA), comparative fit index (CFI), and normed fit Index (NFI) were used to evaluate the fit of the SEM model (66–68).

## Results

### Descriptive Statistics

Before the stay-at-home orders, on average, participants worked from home 1.66 days per week. 61 participants (40.67%) did not have any WFH experiences before the stay-at-home orders. During stay-at-home orders, the average WFH frequency was 4.47 days per week, which was close to the full 5-day WFH per week. 115 participants (76.67%) reported that they worked from home at least 5 days per week. The results highlighted that the stay-at-home orders created rapid and broad shifts to WFH, and WFH was a new experience for most workers. 43 participants (28.67%) indicated that they would like full WFH in the future (after stay-at-home orders), and on average, participants reported that they want to work from home 2.15 days per week.

Table 2 shows the descriptive statistics and correlations among all study variables. Overall, WFH had statistically significant positive correlations with spending time with family members (*r* = 0.56, *p* < 0.01), balancing work and life (*r* = 0.26, *p* < 0.01), and family relationship quality (*r* = 0.27, *p* < 0.01). Age had a statistically significant negative correlation with balancing work and life before WFH (*r* = −0.19, *p* = 0.04), while no significant correlation with the adaptive processes was identified during WFH. Marital status was negatively related to spending time with family members (*r* = −0.18, *p* = 0.04) and family relationship quality (*r* = −0.18, *p* = 0.04) before WFH, but the correlations were not statistically significant during WFH. Education level was not statistically significant with neither of the adaptive processes before WFH, but it had a statistically significant negative correlation with spending time with family members during WFH (*r* = −0.18, *p* = 0.03). Gender was not significantly related to other variables. In addition, the two adaptive processes had a statistically significant positive relation both before WFH (*r* = 0.62, *p* < 0.01) and during WFH (*r* = 0.43, *p* < 0.01). Spending more time with family members was positively related to balancing work and life. The two adaptive processes were also positively related to family relationship quality during both before and during WFH, and the correlations were stronger before WFH (*r* = 0.54, *p* < 0.01; *r* = 0.50, *p* < 0.01) than during WFH (*r* = 0.30, *p* < 0.01; *r* = 0.37, *p* < 0.01). In addition, age had a statistically positive correlation with marital status both before WFH (*r* = 0.35, *p* < 0.01) and during WFH (*r* = 0.39, *p* < 0.01).

**Table 2:** Mean, standard deviations, and correlations of all study variables.

**Variables**	**Mean**	**SD**	**1**	**2**	**3**	**4**	**5**	**6**	**7**	**8**
1. WFH	0.54	0.50	–	0.03	0.02	−0.03	−0.02	0.56^**^	0.26^**^	0.27^**^
2. Gender	0.25	0.43	0.03	–	−0.09	0.00	−0.08	−0.09	−0.16	−0.06
3. Age	42.02	13.64	0.02	−0.10	–	0.35^**^	−0.02	−0.16	−0.19^*^	−0.15
4. Marital status	0.70	0.45	−0.03	−0.10	0.39^**^	–	−0.02	−0.18^*^	−0.17	−0.18^*^
5. Education level	5.18	0.88	−0.02	−0.13	0.02	0.02	–	0.08	0.03	0.04
6. Spending time with family members	3.46	1.28	0.56^**^	0.03	0.09	0.04	−0.18^*^	–	0.62^**^	0.54^**^
7. Balancing work and life	3.40	1.07	0.26^**^	−0.05	0.16	−0.01	−0.16	0.43^**^	–	0.50^**^
8. Family relationship quality	3.70	0.96	0.27^**^	0.04	0.00	−0.02	−0.14	0.30^**^	0.37^**^	–

*Based on N = 150 participants and 263 responses. The upper triangular matrix shows the correlation before WFH, while the lower triangular matrix shows the correlation during WFH. But for the correlations between WFH and other variables, the data covers both before and during WFH. WFH was dummy coded (0 indicates before WFH, while 1 indicates during WFH). Gender was dummy coded (0 indicates women, while 1 indicates men). Marital status was dummy coded (0 indicates single, while 1 indicates married or lived together). SD = Standard Deviation*.

* *p < 0.05,*

** *p < 0.01*.

### Path Analysis

**Figure 2:**
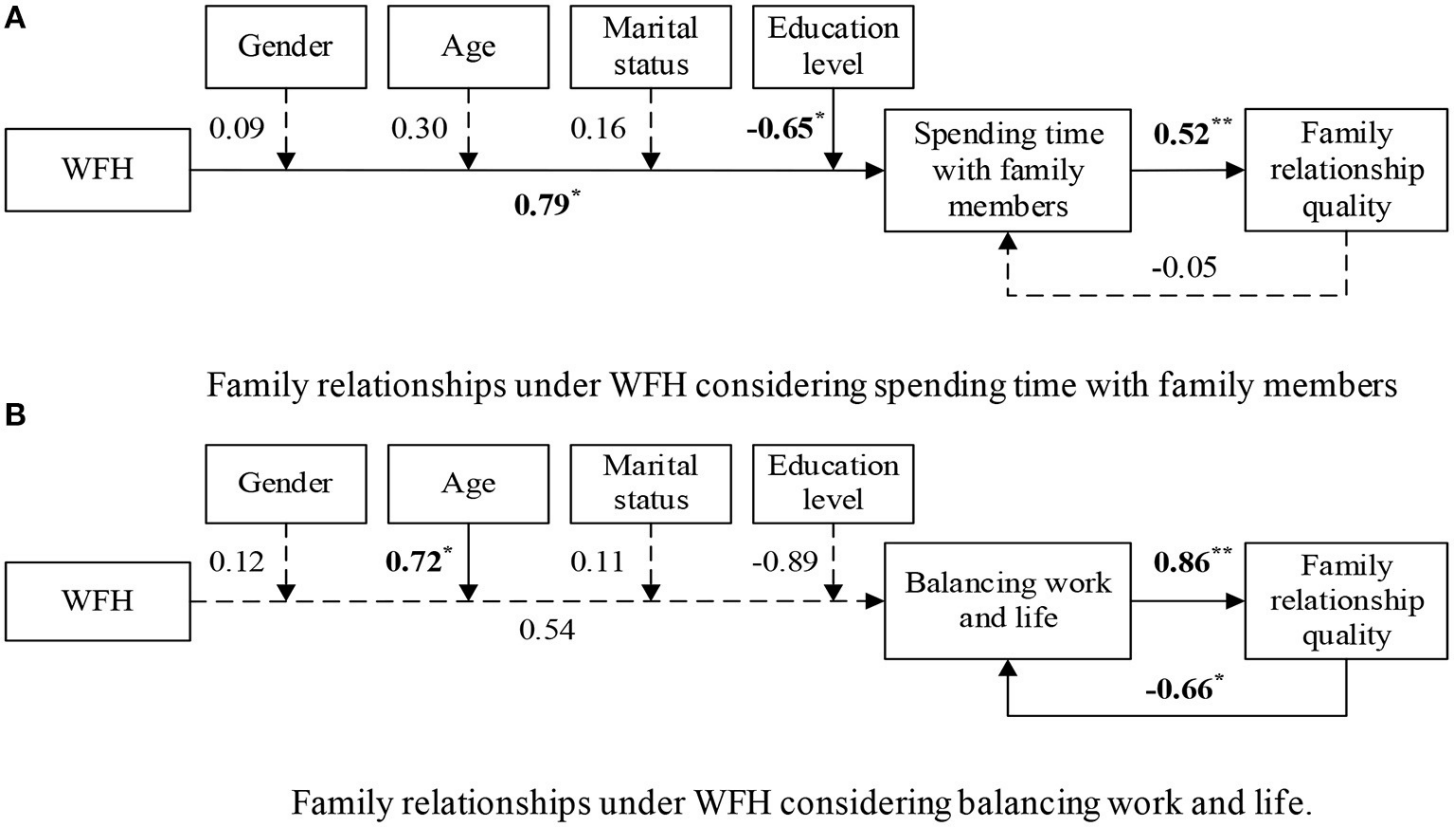
Path models with standardized coefficients. **(A)** Family relationships under WFH considering spending time with family members. **(B)** Family relationships under WFH considering balancing work and life. Both figures were based on *N* = 150 participants and 263 responses. WFH was dummy coded (0 indicates before WFH, while 1 indicates during WFH). Gender was dummy coded (0 indicates women, while 1 indicates men). Marital status was dummy coded (0 indicates single, while 1 indicates married or lived together). Solid arrow lines indicated the statistically significant relations, while dotted arrow lines showed the relations that were not statistically significant. ^*^p < 0.05. ^**^p < 0.01.

Two path models were used to study the two adaptive processes separately^2^. Model 1 focused on the adaptive process of spending time with family members, while Model 2 considered the adaptive process of balancing work and life. Both models exhibited good model fit, as shown in Table 3. Table 4 shows the parameter estimates of the two models, which are also summarized in Figures 2A,B.

**Table 3:** The goodness of fit indices of the structural equation models.

**Indicators**	**Model 1: spending time with family members**	**Model 2: Balancing work and life**	**Recommendation value (66, 67)**
Chi-square	3.12	5.78	-
Degree of freedom (*df*)	8	8	-
$\frac{\chi^{2}}{df}$	0.39	0.72	$0\leq \frac{\chi^{2}}{df}\leq 2$
Standardized RMR (SRMR)	0.01	0.01	0 ≤ SRMR ≤ 0.05
Goodness of fit index (GFI)	1.00	1.00	0.95 ≤ GFI ≤ 1.00
RMSEA estimate	0.00	0.00	0 ≤ RMSEA ≤ 0.05
Comparative fit index	1.00	1.00	0.97 ≤ CFI ≤ 1.00
Normed fit index (NFI)	1.00	1.00	0.95 ≤ NFI ≤ 1.00

**Table 4:** Parameter estimates of the structural equation models.

**Path**			**Unstandardized estimates**	**Standardized estimates**	**Standard error**	***t*** **value**	***p* value**
**Model 1: Spending time with family members**							
WFH	→	Spending time with family members	2.02	0.79	0.36	2.21	0.03
Age	→	Spending time with family members	−0.01	−0.11	0.08	−1.29	0.20
Marital status	→	Spending time with family members	−0.39	−0.14	0.08	−1.67	0.09
Gender	→	Spending time with family members	−0.27	−0.09	0.08	−1.17	0.24
Education level	→	Spending time with family members	0.09	0.06	0.08	0.83	0.41
WFH*Age	→	Spending time with family members	0.02	0.30	0.19	1.56	0.12
WFH* Marital status	→	Spending time with family members	0.43	0.16	0.12	1.38	0.17
WFH*Gender	→	Spending time with family members	0.32	0.09	0.08	1.05	0.29
WFH*Education level	→	Spending time with family members	−0.31	−0.65	0.31	−2.08	0.04
Spending time with family members	→	Family relationship quality	0.39	0.52	0.09	6.04	<0.01
Family relationship quality	→	Spending time with family members	−0.07	−0.05	0.10	−0.51	0.61
**Model 2: Balancing work and life**							
WFH	→	Balancing work and life	1.16	0.54	0.58	0.94	0.35
Age	→	Balancing work and life	−0.02	−0.22	0.14	−1.54	0.12
Marital status	→	Balancing work and life	−0.47	−0.20	0.13	−1.48	0.14
Gender	→	Balancing work and life	−0.49	−0.20	0.13	−1.56	0.12
Education level	→	Balancing work and life	0.04	0.03	0.12	0.27	0.79
WFH*Age	→	Balancing work and life	0.03	0.72	0.33	2.17	0.03
WFH* Marital status	→	Balancing work and life	0.23	0.11	0.19	0.56	0.58
WFH*Gender	→	Balancing work and life	0.36	0.12	0.13	0.88	0.38
WFH*Education level	→	Balancing work and life	−0.36	−0.89	0.53	−1.70	0.09
Balancing work and life	→	Family relationship quality	0.78	0.86	0.15	5.57	<0.01
Family relationship quality	→	Balancing work and life	−0.73	−0.66	0.34	−1.96	0.05

Hypothesis 1 examined the relation between WFH and the adaptive processes. The results showed that WFH had a statistically significant positive relation with spending time with family members (path coefficient = 0.79, *p* = 0.03). However, the relation between WFH and balancing work and life was not statistically significant (*p* = 0.35). Thus, Hypothesis 1 was only supported for the adaptive process of spending time with family members.

Hypothesis 2 proposed that demographic differences moderate the relation between WFH and adaptive processes. The findings showed that education level had a statistically significant negative moderating effect on the relation between WFH and spending time with family members (path coefficient = −0.65, *p* = 0.04). That is, workers with higher education levels tended to spend less time with family members during WFH. Age had a statistically significant positive moderating effect on the relation between WFH and balancing work and life (path coefficient = 0.72, *p* = 0.03). That is, older workers were able to better balance work and life under WFH. However, gender and marital status did not statistically significantly moderate the relations between WFH and either of the adaptive processes. Therefore, Hypothesis 2 was partially supported.

Hypothesis 3 suggested that the two adaptive processes positively associate with family relationship quality. Results showed that both spending time with family members (path coefficient = 0.52, *p* < 0.01) and balancing work and life (path coefficient = 0.86, *p* < 0.01) had statistically significant positive associations with the family relationship satisfaction. Thus, Hypothesis 3 was supported.

Finally, Hypothesis 4 assumed that family relationship quality, in turn, predicts the adaptive processes. The results suggest that family relationship satisfaction had a negative relation with balancing work and life (path coefficient = −0.66, *p* = 0.05). However, family relationships did not have a statistically significant relation with spending time with family members. Thus, Hypothesis 4 was not for spending time with family members but was for the adaptive process of balancing work and life, in agreement with there being a reciprocal relation between balancing work and life and family relationship quality.

## Discussion

### General Findings

In general, the results of the current study partially supported the proposed conceptual model on the relations among WFH, demographic differences (vulnerabilities), adaptive processes, and family relationship quality. WFH was positively related to the perceived time spent with family members, which then positively associated with family relationship quality. Workers with lower education levels tended to spend more time with family members during WFH. WFH did not statistically significantly relate to the perceived work-life balance overall, yet older workers tended to have a better balance between work and life under WFH (as compared to before WFH). Balance in work and life contributed to improved satisfaction toward family relationships, yet family relationship quality, in turn, negatively predicted the balance between work and life. We discuss these findings in detail below.

First, WFH had a positive association with spending time with family members. This could be due to WFH eliminating the need to commute to the office, allowing workers to spend more time at home (19, 28, 29, 33). Workers can benefit from the saved traveling time and flexible schedules to spend more time accompanying their family members. Also, the WFH experiences during the COVID-19 pandemic led to social isolation and loneliness (16, 69) that stimulated individuals to spend more time with family members to get social support (9, 70). The qualitative comments provided by the participants support the results, as shown in Table 5.

**Table 5:** Summary of sample comments from participants.

**Key points**	**Sample comments from participants**
WFH positively contributed to spending time with family members.	“I am enjoying time at home with family. It is a much desired and appreciated the time to be with family and not work all the time.”
	“The time was gained without spending time for travel.”
	“There is no need to commute, saving about 2 hours per day.”
WFH is positively associated with work-life balance.	“With home-based work, I am saving time getting dressed, commuting, fewer distractions. I also save time not having to attend unnecessary meetings. I am also able to exercise using the saved time.”
	“I feel less exhausted when working at home. Besides, I eat healthier at home. I have time for meditation which improves productivity.”
WFH is negatively associated with work-life balance.	“Work is interference with private life, especially family matters.”
	“Having my work be a part of my home life tends to blur the boundaries between work and life.”
	“…more distractions from family members, especially children.”
	“…more housework: parenting, child education, cleaning and sanitizing, etc.”
There is a tradeoff between family relationships and work-life balance.	“More distractions/responsibilities at home impact my work productivity.”
	“The demands of parenting tend to take precedent overwork during normal business hours.”
	“Parenting and cooking influence work productivity.”

However, the relation between WFH and balancing work and life was not significant. It suggests that work-life balance did not change significantly when shifting to WFH. The null finding of the current study is consistent with a recent study suggesting that remote work does not have a significant association with work-life balance (58). This might be because WFH is both positively (71, 72) and negatively associated with work-life balance (28, 41, 73), which, overall, is reflected in the results as a null relation between WFH and work-life balance. This was supported by the qualitative comments from the participants. Some participants expressed positive impacts of WFH toward work-life balance, whereas other participants reported difficulties in balancing work and life, which are summarized in Table 5.

Second, education level moderated the relation between WFH and spending time with family members. Under WFH, participants with lower education levels reported spending more time with family members but those with higher education did not. One possible reason is that individuals with higher education levels tend to have more work burdens and are responsible for fewer family responsibilities (49). Thus, they tend to spend less time with family members even during WFH. Age moderated the relation between WFH and balancing work and life. Under WFH, older workers reported experiencing better work-life balance than younger workers. This might be because older workers have more experience in balancing work and life, and thus tend to be better at maintaining work-life balance (47). They also have higher levels of autonomy in work, which can buffer their work stress and reach better work performance (51). Older workers also tend to have better social supports and family embeddedness (52), which further helps them to better balance work and life. Gender and marital status were not significant moderators. Another study also identified that gender was not a significant moderator (43). In addition, the non-significant roles of gender and marital status might be due to a limitation of the data in the study, which is that men and married individuals accounted for the majority of participants.

Third, the two adaptive processes—spending time with family and balancing work and life—were effective responses to WFH that positively related to family relationship quality. Family is a critical part of life (30). The balance between work and life enables individuals to perform both work and life roles (74, 75), especially family responsibilities, which contributes to better family relationships. In addition, interactions with family members during WFH provide individuals with social support and interpersonal interactions (9, 16), which then improve family relationships and ensure physical and psychological wellbeing (13, 27). The results suggested that balancing work and life contributed more toward family relationship quality than spending time with family members. This might be because work-life balance focuses on the relative balance between work and life, while spending time with family members only considers the absolute amount of time devoted to family/life aspects. The former is more likely to contribute to overall wellbeing (30), enhancing family relationships. In addition, the findings suggested that the two adaptive processes were positively correlated. Thus, the two adaptive processes facilitated each other to improve family relationships during WFH.

Finally, there was a tradeoff between family relationship quality and work-life balance because family relationship quality, in turn, negatively predicted the perceived work-life balance. This might be because better family relationships require more resources (e.g., energy, attention) for family (27), which could limit attention allocated to work, making it difficult to establish a work-life balance (76). Previous research found that distraction from family members and performing housework and childcare tasks are the major obstacles to work during WFH (77). This part was also supported by comments from participants shown in Table 5. Thus, considering the tradeoff, while maintaining strong family relationships, family members should be mindful of the members' work needs and provide space for work-related tasks. Doing so could help family members establish a balance between work and life, contributing to a positive cycle that could then help improve family relationships.

### Theoretical and Practical Implications

Theoretically, the current study contributes to our understanding of family relationships, especially the VSA model, under a rapidly growing work condition—WFH. The study identified specific adaptive processes that could help improve family relationship quality under WFH. The findings support the effectiveness of interaction and communication (e.g., spending time with family members) and work-life balance (e.g., balancing work and life) on improving family relationships. In addition, the findings supported a new insight into the benefits of the WFH arrangement from the family relationship perspective. Although WFH created various challenges for workers and families, it also has a positive association with family relationships, given proper adaptive processes. Vulnerabilities (demographic differences, e.g., education level and age) were statistically significant factors moderating the effects of adaptive processes on WFH. Overall, the current study advances the understanding of family relationships under WFH considering proper adaptive processes and individual characteristics.

From a practical perspective, the findings of the current study provide three suggestions for improving family relationships under WFH (both during the COVID-19 pandemic and other situations). First, it is recommended for individuals to spend more time with family members during WFH, which is positively associated with family relationships. This is especially the case for highly educated workers, who tended to report spending less time with family members during WFH. Strategies to increase the interaction with family members include fostering communication with them (44), developing a positive family atmosphere (78), and investing more time and attention in homeschooling (29). Second, strategies should be provided to younger workers who perceived worse work-life balance during WFH. Not working extended hours (31), improving time and stress management skills (31, 79), creating a positive work environment by fostering collegiality, enhancing open communication, and achieving mutual respect (79) are all useful practices for improving the balance between work and life. Third, combining both effective adaptive processes, individuals could strive toward building a home-centered work-life balance to ensure the time spent with family members while balancing work and life (30) by allocating more time and energy to family life while maintaining basic work-life division (76). One potential method to achieve this goal is by developing family-friendly work strategies (35) with a specific focus on WFH arrangements. Family-friendly work strategies emphasize that work should facilitate the reconciliation of work and family life, and includes practical help with childcare, changes of work schedule according to family needs, and relevant information and training on balancing work and family life (80). These strategies help individuals better adapt to WFH and improve family relationships.

### Limitations and Future Directions

The current study has some limitations, which also suggest several future directions. First, the study did not examine specific forms of family relationships, such as marital relationships, parental relationships, and intergenerational relationships (12), and findings might vary across these different forms of family relationships. For example, marital relationships and parent-adolescent relationships could face different challenges (81). Future studies should examine the specific types of family relationships to identify targeted strategies that work for different relationships. Second, all measures were self-reports, and the study relied on participants' recall on the study variables before WFH. Self-reports may be influenced by extraneous factors (82), and the variability of recall ability may influence the quality of data (83). The adaptive processes and family relationship quality might also vary across different time points throughout WFH. Future work can apply longitudinal methods to explore the impacts of WFH on family relationships. Third, the analyses are based on correlations of the self-report measures, which restricts the strength of the cause-effect conclusions.

A fourth limitation is that the study only covered a limited number of variables in the VSA model, while future studies can explore other forms of stress, vulnerabilities, and adaptive processes to expand the understanding of WFH and family relationships. The vulnerabilities examined in the current study only included stable demographic indicators, while other psychological or social indicators were not examined. For example, personality traits (e.g., impulsivity, aggressiveness, and negative affectivity) are important factors that influence close relationships, including chronic stress related to partners (21, 24, 55, 63). Mental health (e.g., depression and anxiety) also impact the interaction with others (22, 46, 84) and the balance between work and life (32). During the COVID-19 pandemic, individuals experienced more mental health issues, which may influence their social connection and adaptive process within family relationships (16). In addition, factors such as family size and the number of children or elderly family members (12, 43) influence the content and complexity of family responsibilities (53), which may further impact adaptive processes and family relationships. Considering different vulnerabilities may help understand how diverse groups of individuals adapt to WFH and how it impacts family relationships.

Moreover, the current study only considered the stress associated with adjusting to the WFH arrangement (at the early stage of the pandemic), while there are many other potential sources of stress. For example, during the global pandemic, being infected with COVID-19 is a major stress for the public (85), and movement restriction due to stay-at-home orders could be a source of stress leading to reduced social support and increased loneliness (69). Exploring different stressful events would contribute to a more comprehensive understanding of the individual's adaptive processes and how they influence family relationships. In addition, the current study examined two adaptive processes that were included in the model: spending time with family members and balancing between work and life. While these are adaptive processes closely related to family relationships, more processes could be examined in the future, including effective communication with family members (18) and taking more household responsibilities (12). Examination of multiple adaptive processes may help identify strategies and recommendations for individuals to better adapt to WFH arrangements.

Work characteristics are also critical factors that should be examined in future studies. The type of industry was examined in a supplementary analysis but did not find statistically significant effects. However, as the data collection did not specifically focus on work characteristics, the participants' industry types in the current data collection might not be representative. Future studies should examine a wide range of industry types. Moreover, there are some other factors. For example, job control and demands (86), employment type (self-employed or employed by others) (87), and task identity and significance (88) significantly influence work-life balance. In addition, people with different types of occupations (77, 89), autonomy (40), and work environment (40) have different WFH experiences. Future studies can explore these factors' effects on family relationships.

In addition, the study measured each adaptive process and family relationship quality using single items. Future studies can apply more comprehensive matrixes to measure the variables. Finally, the distribution of educational status in the current sample deviated from the general population, and men and married individuals accounted for the majority of participants in the study. The findings of the current study could be further examined with a sample representing the educational distribution of the general population.

## Conclusion

An unprecedented practice of WFH caused by the COVID-19 pandemic created sudden and broad changes for both workers and their families, which further influenced family relationships. This study examined the impacts of WFH on family relationships through two adaptive processes (i.e., spending time with family members and balancing work and life) while considering the moderating effect of individual differences. Results suggest that WFH improved family relationships through proper adaptive processes, and the relations were moderated by the education level and age of individuals. Specifically, workers spent more time with family members during WFH, which was positively associated with family relationship quality. During WFH, workers with higher education levels reported spending less time with family members and faced more challenges in maintaining family relationships. In addition, although balancing work and life was not statistically significantly associated with WFH, the adaptive process was positively related to family relationships. During WFH, younger workers faced more challenges than older workers in balancing work and life and maintaining family relationships. The study provides a novel insight into the understanding of how individuals adapt to WFH and how such adaptive processes impact their family relationships. The findings also provide practical suggestions to help workers with different characteristics (e.g., gender, age, marital status, education) achieve better family relationships during WFH.

## Data Availability Statement

The data presented in this study will be available on request from the corresponding author. The data are not publicly available due to the privacy of all participants.

## Ethics Statement

The studies involving human participants were reviewed and approved by Purdue's Institutional Review Board, Office of the Executive Vice President for Research and Partnerships, Purdue University. Written informed consent for participation was not required for this study in accordance with the national legislation and the institutional requirements.

## Author Contributions

HW and YC: conceptualization. HW and QS: methodology. HW: formal analysis, investigation, and writing—original draft preparation. QS, YC, and RP: writing—review and editing. All authors contributed to the article and approved the submitted version.

## Funding

This work was funded by the School of Construction Management Technology, Purdue Polytechnic Institute, Purdue University.

## Conflict of Interest

The authors declare that the research was conducted in the absence of any commercial or financial relationships that could be construed as a potential conflict of interest.

## Publisher's Note

All claims expressed in this article are solely those of the authors and do not necessarily represent those of their affiliated organizations, or those of the publisher, the editors and the reviewers. Any product that may be evaluated in this article, or claim that may be made by its manufacturer, is not guaranteed or endorsed by the publisher.

## Footnotes

^1^The number of responses is: 150 × 2 − 27 − 8 − 2 = 263, where 27, 8, and 2 are the number of removed responses.

^2^A helpful reviewer suggested to examine work characteristics (e.g., type of industry) in addition to the demographic variables examined in the current analysis. A supplementary analysis was conducted to examine the relations among WFH, type of industry, two adaptive processes, and family relationship quality. Results suggested that the main effect and the moderating effect of type of industry on the relation between WFH and spending time with family members were both statistically not significant (*p* = 0.69 and 0.32). That is, how the participants adapted to WFH did not statistically significantly differ across different types of industries. Then, the model for “balancing work and life” exhibited poor model fit.
